# Development of a heat labile antibiotic eluting 3D printed scaffold for the treatment of osteomyelitis

**DOI:** 10.1038/s41598-020-64573-5

**Published:** 2020-05-05

**Authors:** Ji-Hyun Lee, Jong-Min Baik, Young-Soo Yu, Joo Hyun Kim, Chi Bum Ahn, Kuk Hui Son, Joo-Hyung Kim, Eun Seok Choi, Jin Woo Lee

**Affiliations:** 1 Department of Molecular Medicine, College of Medicine, Gachon University, Incheon, Republic of Korea; 2 Department of Orthopedic Surgery, Gil Medical Center, Gachon University College of Medicine, Incheon, Republic of Korea; 3 Department of Health Sciences and Technology, GAIHST, Gachon University, Incheon, Republic of Korea; 4 Department of Thoracic and Cardiovascular Surgery, Gil Medical Center, Gachon University College of Medicine, Incheon, Republic of Korea; 5 Department of Mechanical Engineering, Inha University, Incheon, Republic of Korea; 6 Department of Orthopedic Surgery, Chungnam National University Hospital, Daejeon, Republic of Korea

**Keywords:** Non-model organisms, Microbiology techniques, Translational research

## Abstract

In general, osteomyelitis is treated with antibiotics, and in severe cases, the inflammatory bone tissue is removed and substituted with poly (methyl methacrylate) (PMMA) beads containing antibiotics. However, this treatment necessitates re-surgery to remove the inserted PMMA beads. Moreover, rifampicin, a primary heat-sensitive antibiotic used for osteomyelitis, is deemed unsuitable in this strategy. Three-dimensional (3D) printing technology has gained popularity, as it facilitates the production of a patient-customized implantable structure using various biodegradable biomaterials as well as controlling printing temperature. Therefore, in this study, we developed a rifampicin-loaded 3D scaffold for the treatment of osteomyelitis using 3D printing and polycaprolactone (PCL), a biodegradable polymer that can be printed at low temperatures. We successfully fabricated rifampicin-loaded PCL 3D scaffolds connected with all pores using computer-aided design and manufacturing (CAD/CAM) and printed them at a temperature of 60 °C to prevent the loss of the antibacterial activity of rifampicin. The growth inhibitory activity against *Escherichia coli (E. coli)* and *Staphylococcus aureus (S. aureus)*, the representative causative organisms of osteomyelitis, was confirmed. In addition, we optimized the rifampicin-loading capacity that causes no damage to the normal bone tissues in 3D scaffold with toxicity evaluation using human osteoblasts. The rifampicin-releasing 3D scaffold developed herein opens new possibilities of the patient-customized treatment of osteomyelitis.

## Introduction

Osteomyelitis is a bacterial infection of the bones that is common in various pathological conditions and may occur at all ages and in any bone^1^. In general, osteomyelitis is caused by hematogenous dissemination, continuous diffusion from adjacent soft tissues and joints, and direct inoculation due to surgery^1^. Osteomyelitis encourages the growth of bacteria such as *Staphylococcus epidermidis*, *Staphylococcus aureus (S. aureus)*, *Pseudomonas aeruginosa*, *Serratia marcescens*, and *Escherichia coli (E. coli)*^2^. The discovery of penicillin by Fleming in 1928 has led to a rapid decrease in the rate of associated morbidity, mortality, and complications^3^. However, the resistance of *S. aureus*, the major causative organism, to penicillin has increased since 1960, and has affected the treatment and prognosis of osteomyelitis^4,5^. In general, osteomyelitis is treated with antibiotics depending upon the severity of the infection and treatment time; however, the recurrence rate is as high as 30% once the infection has progressed, thereby posing difficulties for disease treatment^6^.

Chronic osteomyelitis is associated with recurrence and is caused by the swelling of the infected tissue, which impedes blood circulation to the affected area and prevents the transfer of antibiotics. In addition, the infected areas that do not receive oxygen and nutrients from the blood undergo rapid necrosis and accelerate bacterial growth. In most severe chronic osteomyelitis cases, debridement is carried out to remove necrotic tissues. However, it is impossible to completely remove bacteria, which are the root cause of osteomyelitis. Therefore, long-term administration of high doses of antibiotics is opted for the complete removal of the bacteria and prevention of recurrence. However, the orally or intravenously administered antibiotic may not be delivered to the infected area and cause complications, owing to a burden on the liver or kidney. In particular, bio-films produced by the bacteria may prevent the effect of antibiotics, thereby making the treatment regimen more complicated^7^.

To date, several studies have been conducted on the local delivery of antibiotics directly to the infected site to overcome the limitations associated with indirect drug delivery^8^. The direct application of drugs or antibiotics to the infected site offers the advantage of prevention of side-effects and reduction of antibiotic usage. Therefore, the strategy of mixing antibiotics with spherical carriers (beads) using poly (methyl methacrylate) (PMMA) has been widely used^9,10^. However, PMMA generates heat (up to 110 °C) during the polymerization process and hence, may be unsuitable with many antibiotics. In addition, the amount of antibiotic eluted from the beads cannot be controlled. As PMMA is non-biodegradable, there is a need for an additional surgery to remove the beads after treatment. Rifampicin, first synthesized in 1965, exhibits excellent antibacterial properties against *Mycobacterium tuberculosis* and exerts a broad spectrum of antibacterial activity against gram-positive and gram-negative bacteria^11^. Recently, PMMA beads loaded with rifampicin were applied in a traumatic musculoskeletal model^12^. However, rifampicin inhibits the polymerization of PMMA and may not be mounted in the form of PMMA beads, although it shows excellent efficacy in osteomyelitis treatment.

Three-dimensional (3D) printing is a technique used for the production of a desired three-dimensional shape by stacking sectional shapes based on two-dimensional sliced digital data^13–18^. In particular, the combination of 3D printing with medical imaging data, as observed with computed tomography (CT) and magnetic resonance imaging (MRI) with a reverse engineering technology, facilitates the development of a customized construct to the affected areas in patients. In addition, the fused deposition modeling (FDM)-based 3D printing technology allows for the construction of a structure at low temperatures by the selection of the printing material as the carrier^19^. As the process of polymerization is excluded, this method allows the use of several different antibiotics without any restriction. Furthermore, it also controls the drug release amount through the free designing of the external and internal shapes of the structure. In particular, the use of a biodegradable polymer as a printing material may avoid the need to perform a second operation to remove the implanted structure after the treatment of osteomyelitis, unlike the PMMA beads.

Among the various biodegradable biopolymers, polycaprolactone (PCL) has a low melting temperature and allows the progression of the 3D printing process at low temperatures^20^. This phenomenon may facilitate the loading of heat-labile antibiotics. In addition, unlike poly (lactic acid) (PLA) and Poly(D,L-lactic-co-glycolic acid) (PLGA), PCL does not produce acidic by-products during the biodegradation process, thereby causing less damage to the surrounding tissues^21,22^. So far, PCL has been used as a biomaterial for the reconstruction of various tissues such as bone, cartilage, liver, muscle, vessel, and ligament in regenerative medicine^23–27^.

In the present study, we have developed a drug-releasing scaffold with rifampicin that proved effective for biofilm removal but could not be used with the conventional PMMA beads using FDM-based 3D printing system and PCL. We confirmed the feasibility of the customized treatment strategy for osteomyelitis by evaluating the bacterial growth inhibition ability of the developed scaffold in the representative bacterial environment of osteomyelitis.

## Results

### Scaffold fabrication by 3D printing and characterization

**Figure 1:**
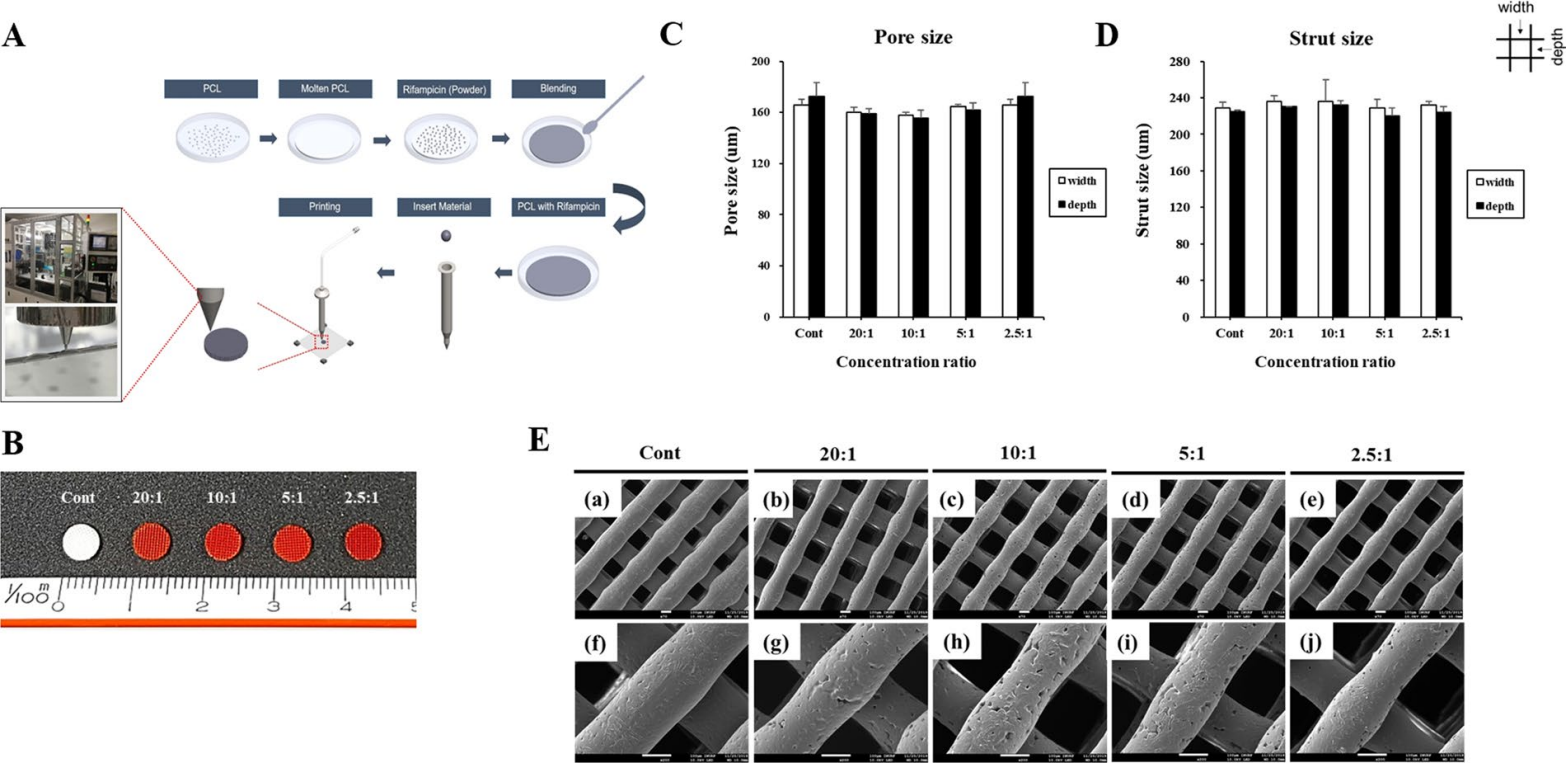
Fabrication of a rifampicin-loaded 3D scaffold. (**A**) Schematic illustration of the procedure involved in the fabrication of the rifampicin-loaded scaffold using our 3D printing system. (**B**) Photographs of the rifampicin-loaded scaffold at different concentrations.. Electron micrographs were analyzed using microscope to determine the (**C**) pore size and (**D**) strut size of the scaffolds. (**E**) SEM image of scaffolds with rifampicin concentration of (a,f) Cont, (b,g) 20:1, (c,h) 10:1, (d,i) 5:1, and (e,j) 2.5:1. The image of (f–j) indicate magnified view of images of (a–e), respectively. a–e: x70, f–j: x200, scale bar: 100 um.

To determine the feasibility of the heat-labile rifampicin-loaded scaffolds, the 3D scaffolds were designed and printed as described in Materials and Methods (Fig. 1A). Scaffolds with two-layer patterns of the strands were plotted and processed. Scaffolds with two-layer patterns of the strands such as 0/90 lattice structure were plotted and processed. The size of the scaffold for *in vitro* experiments was 5 × 5 × 1 mm. The addition of rifampicin and PCL resulted in the change in the color of the scaffold to orange owing to the color of the drug, and the color intensity was proportional to the concentration of rifampicin (Fig. 1B). The shape and accuracy of the fabricated scaffolds were evaluated at each stage as well as after the completion of fabrication. As shown in Fig. 1C,D, the pore size and strut size were measured using an optical microscope to confirm that no special changes were observed in the strand and scaffold shapes. The scaffolds were fabricated layer by layer, and the homogeneous porous structure was confirmed by morphology of scaffolds using SEM (Fig. 1E).

### Drug release profiles

**Figure 2:**
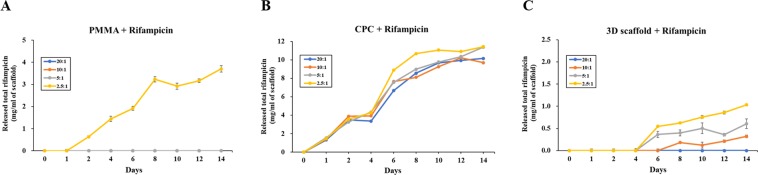
The release curve of rifampicin from scaffolds at various loading concentrations and materials for 14 days. (**A**) R-PMMA group; (**B**) R-CPC group; (**C**) R-3D scaffold group. R: rifampicin, PMMA: poly (methyl methacrylate), CPC: calcium phosphate cement.

The drug release profiles of the rifampicin-loaded scaffolds were evaluated under physiological conditions (37 °C and pH 7.4). The release curve was expressed as the cumulative mass of rifampicin per mass of scaffold to compare different materials and concentration conditions. In the case of rifampicin-mixed PMMA scaffold (R-PMMA), rifampicin was only detected in the sample prepared at 2.5:1 ratio (Fig. 2A). The rifampicin-mixed CPC scaffold (R-CPC) showed a rapid rifampicin release profile from 4 to 6 days. The release of rifampicin was not dependent upon the concentration of rifampicin in the sample, and only the 2.5:1 ratio group showed a slight increase in the release of rifampicin (Fig. 2B). Rifampicin was released from each sample prepared from PCL scaffold using 3D printing system (R-3D scaffold) (Fig. 2C). Rifampicin was completely released from all the scaffolds within 14 days. Thus, only the R-3D scaffolds showed a consistent release profile of rifampicin, and this effect was suitable for bone infection treatment.

### Antibacterial activity assay

**Figure 3:**
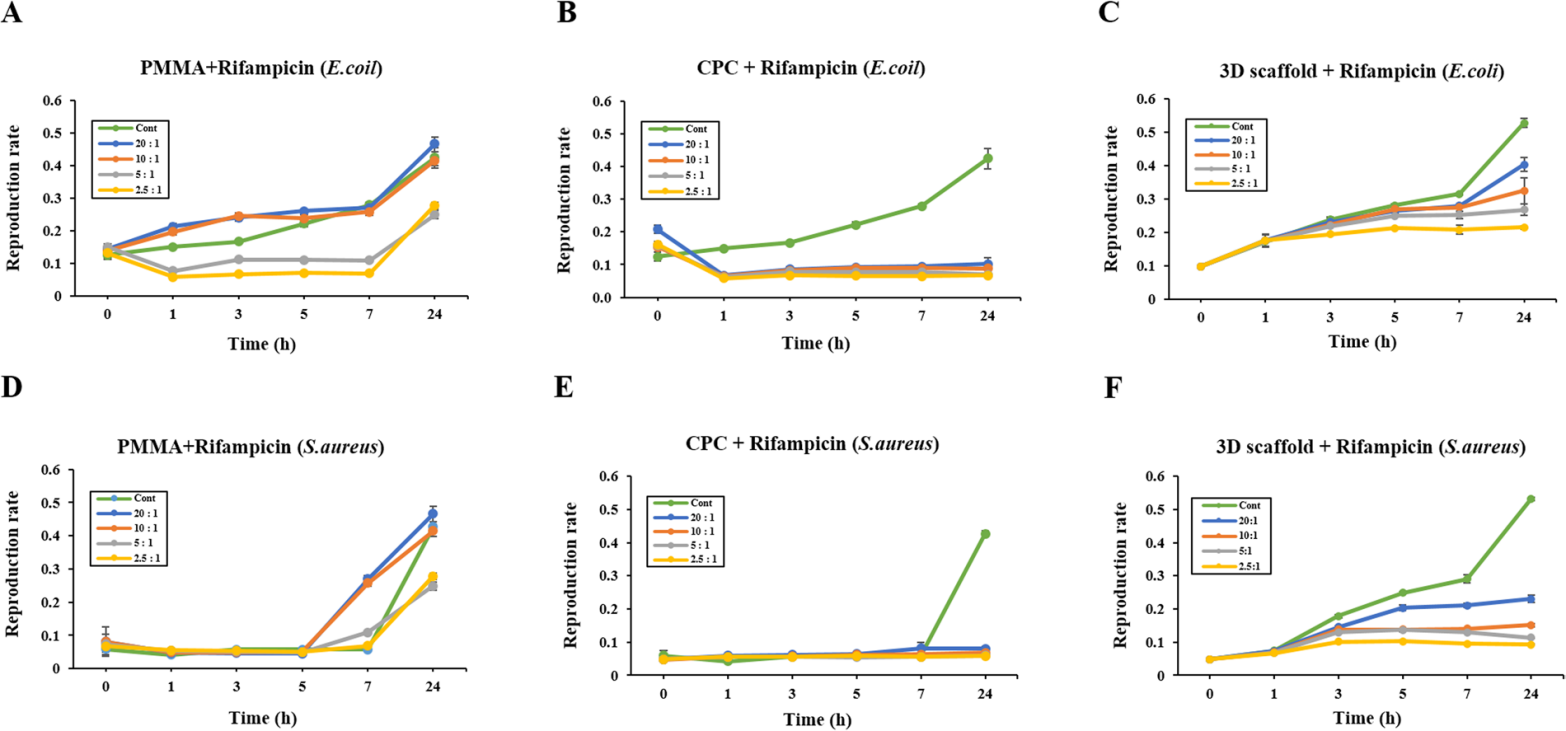
Antibacterial activity with the broth dilution assay. (**A**) R-PMMA group; (**B**) R-CPC group; (**C**) R-3D scaffold group. Bacterial count was monitored at 0, 1, 3, 5, 7, and 24 h after treatment with the rifampicin-loaded scaffold in *E. coli*. (**D**) R-PMMA group; **(E**) R-CPC group; (**F**) R-3D scaffold group. Bacterial count was monitored at 0, 1, 3, 5, 7, and 24 h after treatment with the rifampicin-loaded scaffold in *S. aureus*. Results are the representative of three independent trials, each performed in triplicates.

To explore the biological efficiency of the scaffolds, we tested their antibacterial activities against *E. coli* and *S. aureus*. Figure 3 show the bacterial inhibition rate in the presence of different scaffolds at various time points. Although R-PMMA group exhibited bacterial inhibitory activity from 1 to 5 h, the effect was significantly lower at 7–24 h (Fig. 3A,D). In contrast, the growth of *E. coli* and *S. aureus* was completely inhibited upon incubation with R-CPC and R-3D scaffolds for up to 24 h (Fig. 3B,E). As shown in Fig. 3C,F, the inhibitory effect on the growth of *E. coli and S. aureus* were same at both 5:1 ratio and 2.5:1 ratio. However, although the growth inhibitory of *E. coil* was reduced in low-concentration, it was confirmed that the inhibitory of the *S. aureus* was maintained.

### Analysis of HOB proliferation

**Figure 4:**
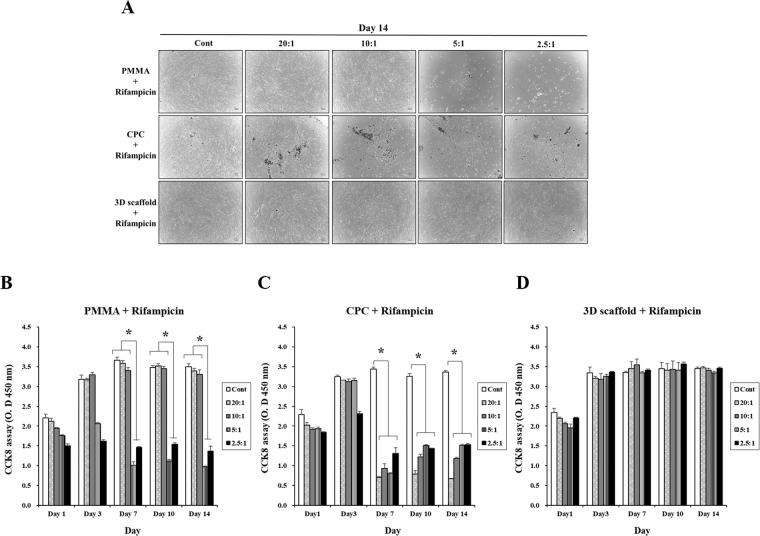
*In vitro* toxicity assay. HOBs (3 × 10^5^) were plated and cultured for 14 days. (**A**) Morphology of HOBs incubated with R-PMMA, R-CPC and R-3D scaffold groups (×4) scale bar: 100 um. (**B**) Effects of R-PMMA, R-CPC and R-3D scaffold groupson HOB proliferation. HOB: human osteoblast cells (*denotes statistically difference, *P < 0.05).

To determine the cell toxicity of the scaffolds, we tested the proliferation of HOBs with the CCK8 assay and evaluated cell morphology for 14 days in each group. As shown in Fig. 4A,B, the proliferation of HOBs in low concentration groups was much higher on day 3 to day 14 than on day 1. However, rifampicin decreased the viability of cells in a dose-dependent manner, indicating that the overdose of R-PMMA groups (5:1 and 2.5:1 ratio) had a negative effect on HOB proliferation. In the R-CPC groups, we showed that cell proliferation was inhibited from day seven in each group except for the control group (Fig. 4A,C). In Fig. 4A,D, the cell number increased continuously up to 3 day in each ratio group. However, the cell proliferation rate was similar among groups at each time point. The results indicated that the cell viability and proliferation of HOBs on scaffolds were not affected by R-3D scaffolds.

## Discussion

The present study developed rifampicin-load scaffold by using the 3D printed with melted PCL at low temperature and also provided that manufacture of heat labile antibiotics could be possibility of osteomyelitis treatment via enhanced of antibacterial activity. Rifampicin-loaded scaffold manufactured using 3D printing possessed a porous structure that has a large surface area and controllable surface and interior features, those allowed it to control the rate and duration of antibiotics unlike the existing bone cement structures using PMMA and CPC. PCL is known to have a melting point of 60 °C and that is lower than 140 °C of PLGA and 190 °C of PLLA, so there is less risk of thermal damage to the materials being mixed. It is also reported that unlike PLA and PLGA, they are highly stable in the body because they do not produce acidic component in biodegradations^28^. However, no report has been yet examined a PCL scaffold using heat labile antibiotics for osteomyelitis treatment. This study was the first to confirm the function of the scaffold by printing a 3D scaffold by mixing the heat labile antibiotic rifampicin with PCL (Fig. 1).

When rifampicin was loaded on PMMA, CPC and PCL 3D printed scaffolds, R-PMMA showed an antibiotic elution was not occurred at all concentrations except for 2.5 to 1 concentration (Fig. 2A). R-CPC showed antibiotics were released in all groups irrespective of a concentration of rifampicin and it proved to be inadequate to control the dissolution rate of rifampicin in R-CPC (Fig. 2B). Recent studies have shown that antibiotics-loaded biodegradable polycaprolactone (PCL) scaffold for treatment of osteomyelitis using three-dimensional (3D) printing can be slowly and continuously released^29,30^. We also confirmed that rifampicin was stably eluted from the R-3D scaffold in proportion to the concentration (Fig. 2C).

In the test for the antimicrobial activity of Rifampicin, R-PMMA was found to have very low antibacterial activity after 24 h against *E. coli* and *S. aureus* at all concentration. R-CPC was shown for antimicrobial activity in both E-coli and *S. aureus*, but rifampicin was released in a short time under all conditions. That result corresponded to the initial burst release profile of Fig. 2B. Especially, For *S. aureus*, only the R-3D scaffold showed the antimicrobial activity dependent on the concentration (Fig. 3). This result means that 10:1 was a sufficient antibacterial activity for *S. aureus* at R-3D scaffold and a high-concentration antibiotic of 5:1was required to inhibit the growth of *E. coli*.

We assumed that we could maximize the effectiveness of antibiotics inside our body by loading rifampicin in the 3D scaffold that can remove a bio-film generated by bacteria. *S. aureus* is a typical osteomyelitis causative bacterium known as the main causative bacterium that forms bio-film^31^. Ryan Trombetta *et al*. reported that the effects of rifampicin using CPC-based 3D printing techniques^32^. However, it was not possible to identify the concentration-defendant antimicrobial forces because of experiment result using one limited concentration group. And it was possible to see that the concentration of antibiotics was maintained continuously, because a large amount of antibiotics was initially eluted^33^. Their results showed that the control of the elution rate *in vivo* is difficult and CPC is not suitable as a local delivery material for antibiotics due to burst release. However, because our R-3D scaffold is based on PCL that absorbs water and is degraded, we suggested that the antibiotic release rate and duration can be controlled by adjusting the antibiotic loading level according to the patient’s infection level. And our scaffold has an additional advantage that is that a removal surgery is not required by a biodegradable characteristic of PCL in itself.

We conducted an *in vitro* experiment using human osteoblast (HOB) cells to investigate whether R-3D scaffold affects bone tissue cell during the local anti-biotic therapy. The optimal antibiotic concentration could be investigated by comparing the survival rate of HOB cells according to the dilution concentration of antibiotics. When observed cell morphology, it was found that the concentration of antibiotics in favor of R-PMMA scaffold had little effect on 10:1, but that the concentration from 5:1 resulted in a damage to the HOB cells (Fig. 4A,B). The R-CPC scaffold group has already showed that cell damage was induced from a low concentration of 20:1 (Fig. 4A). In addition, indicated that cell survival rates decreased in proportion to the levels of rifampicin over time and that R-CPC scaffold showed higher cell toxicity than R-PMMA scaffold (Fig. 4C). These data suggest that antibiotics were released much higher in the R-CPC scaffold than in R-PMMA scaffold, possibly causing cell damage. Contrastively, the R-3d scaffold has been confirmed not negative effected to the survival and toxicity of cells regardless of the antibiotic concentration (Fig. 4A,D).

Therefore, 3D printing based rifampicin eluting scaffold manufacturing technique which can control the degree of antibiotic leaching will have a good effect.

## Conclusion

In this study, rifampicin loaded scaffold using 3D printing technique which develop the cold fabrication process of the thermo-sensitive antibiotics demonstrated that the drug’s effectiveness can be maximized by controlling a drug elution for representative bacteria, *S. aureus* and *E. coli*. Especially, by using PCL of biodegradable material as a carrier for delivery, which suggests that our scaffold may not require a removal surgery due to retain biodegradation properties. In addition, experiments at various concentration conditions allowed the optimization of the antibiotic loading levels to the extent possible to treat osteomyelitis while minimizing normal cell damage. Therefore, our developed 3D scaffold may be a new strategy to treat osteomyelitis more efficiently.

## Materials and methods

### Preparation of rifampicin-loaded PCL

PCL (Mw: 45,000; Sigma-Aldrich, St Louis, MO, USA) was used as a base polymer in the 3D printing of an antibiotic-based scaffold. Rifampicin (Tokyo Chemical Industry Co., LTD., Tokyo, Japan) was chosen as the antibiotic to be loaded into the scaffolds for the therapy of osteomyelitis. PCL and rifampicin were blended as follows: PCL granules were fully melted in the dish of 60 °C for 30 min and the molten PCL was mixed with rifampicin powder. Rifampicin and PCL were mixed in different ratios (20:1, 10:1, 5:1 and 2.5:1). The mixture was stirred until all the components were homogeneously mixed using a digital hotplate stirrer (Daihan scientific, Seoul, Korea) with 60 rpm rotating speed of a magnetic bar.

### Manufacturing of the rifampicin-based scaffold

We used an in-house-developed multi-head deposition system as a 3D printer (Geo technology, Incheon, Korea) (Fig. 1A). This system is equipped with a temperature and pneumatic controller with an x-y-z motion controller. Four isolated heads assembled with a heater and syringe were used for the fabrication of hybrid scaffolds in the presence of various combinations of biomaterials. The temperature and pressure of each head were individually controlled up to 300 °C and 800 KPa, respectively. A linear motor, linear encoder, and guide were installed to control x-axis and y-axis motions at an accuracy of 0.5 μm and repeatability of 2 μm. In the z-axis, the accuracy and repeatability were both 5 μm. The working space of this system was designed to be 400 mm × 400 mm × 250 mm to allow fabrication of relatively large volumes of constructs. For the formulation of a drug-releasing scaffold using 3D printing system, Solidworks software (Dassault Systèmes, Paris, France) was used as one of the computer-aided design (CAD) programs. The internal architecture of the scaffold was designed using a mesh shape, and the interconnected lines were orthogonally and cylindrically distributed in the X, Y, and Z directions. A 3D printer was used for disk fabrication (disk dimension: 5 mm (ϕ) × 1 mm). The printing was conducted at 60 °C to maintain the stability of the antibiotic and a pneumatic pressure of 800 kPa. A speed of 100 mm/min was applied to dispense the PCL-antibiotic mixture through a nozzle with an inner diameter of 200 µm.

### Rifampicin-PMMA (or CPC) disk scaffold manufacturing

Rifampicin and PMMA or calcium phosphate cement (CPC) were mixed in different ratios (20:1, 10:1, 5:1 and 2.5:1). For Rifampicin-PMMA and Rifampicin-CPC, rifampicin was mixed PMMA using NOVOSET Cat No. BC100 (CGBio, Seongnam, Korea) and rifampicin was mixed CPC using simplex Bone Cement Cat No. 6191-0-001 (Stryker, Kalamazoo, Michigan, USA) according to the manufacturer’s instructions, respectively. And mixed with rifampicin at different ratios and stir thoroughly for a total of 1–2 minutes. The mixed PMMA or CPC were hardened in a 5 mm disk shaped scaffold frame.

### Bacterial cultures

The stability of the antibiotics in all the samples was tested with the broth dilution method in the presence of appropriate negative controls. *S. aureus* (KCTC No.3881) and *E. coli* (KCTC No. 2571) were used for experiments. Bacterial colonies were selected from agar plates and inoculated into 5 mL Luria Bertani (LB) broth (BD, Franklin Lakes, NJ, USA), followed by overnight incubation on a shaker incubator set at 37 °C and 170 rpm. About 1.5 × 10^8^ colony-forming units (CFUs)/mL were inoculated onto an LB agar plate and incubated overnight at 37 °C.

### Broth dilution assays

LB broths were inoculated with 50 μL of bacterial cultures and treated with specimens from each group. These cultures were incubated for 24 h at 37 °C and 170 rpm on a shaker. Triplicates from each group were tested and compared with controls. The absorbance at 600 nm wavelength was measured with an enzyme-linked immunosorbent assay (ELISA) reader (Soft Max Pro5, Molecular Devices, San Jose, CA, USA).

### Drug release test

Phosphate-buffered saline (PBS; Gibco, Waltham, MA, USA) at pH 7.4 was selected as the release solution in this study to mimic the *in vivo* environment. The concentration of the released drug was measured using a UV spectrophotometric analysis. The rifampicin-based scaffold was soaked in 5 mL PBS at 37 °C and the solution was collected at different time points (1 to 14 days). The amount of rifampicin released was verified by measuring the absorbance at 340 nm wavelength, while the release rate was calculated according to the standard absorbance concentration curve obtained from the rifampicin-containing PBS.

### Cell culture

Human osteoblasts (HOBs, Cat No. C-12720) were purchased from PromoCell (Heidelberg, Germany). The cells were cultured in an osteoblast growth medium containing 10% fetal calf serum (FCS; Promo cell, Heidelberg, Germany) and 100 units/mL of penicillin/streptomycin (P/S; Gibco, Waltham, MA, USA) at 37 °C in a humidified atmosphere containing 5% CO_2_. The medium was changed every 2–3 days. After reaching confluency, the cells were removed from the culture dish using 0.25% trypsin-ethylenediaminetetraacetic acid (EDTA) (Gibco, Waltham, MA, USA), centrifuged, and resuspended in osteoblast growth medium (Cat No. C-27001, Promo cell, Heidelberg, Germany).

### Cell proliferation assay

Before dipping the scaffold in the cell culture dish, the scaffolds were washed for overnight with 70% ethanol, and repeatedly rinsed with ultra-pure water and sterilized by UV light for 30 min. Cell proliferation rate was measured using Cell Counting Kit 8 (CCK8; Dojindo, Kumamoto, Japan). Cells were seeded in a 48-well plate at a density of 3 × 10^5^ cells. And then, rifampicin-loaded PCL 3D scaffolds were added to each well plate. After 1 and 3 days, 100 µL of CCK8 solution was added to each well and the cells were incubated at 37 °C for 2 h. The absorbance at 450 nm wavelength was measured using an ELISA reader (VERSA MAX; Molecular devices, San Jose, CA, USA).

### Statistical analysis

All the experiments were performed in triplicates and the representative or average data were presented, unless otherwise stated. The data were analyzed using Prism (ver. 7; GraphPad software, San Diego, CA, USA) software. The data within a given group or between groups were compared using one-way analysis of variance (ANOVA). Significant difference was defined as *p < 0.05.
